# Obstetrics

**Published:** 1905-07-01

**Authors:** 


### OBSTETRICS.

(*Continued from page* 209.)

**The Toxæmia of Pregnancy.**—Davis[5] draws attention to the importance of examining the blood as well as the urine in cases of toxæmia occurring during pregnancy. With regard to the urine he points out that although albumen is generally present in eclampsia, cases do occur in which the toxicity of the urine is not materially increased and albumin is absent. It is interesting to observe that the chlorides of the urine and the amount of water may be greatly lessened, and leucin is sometimes present. The blood serum is found to be distinctly toxic, and when injected into animals causes convulsions and death. The source of the toxin has not yet been discovered. Modern investigation has shown that the kidneys are but slightly responsible, that the liver is the organ most frequently diseased, and the one which shows the most striking post-mortem changes. Hæmorrhagic necrosis of the liver with multiple thrombosis is the characteristic lesion found. These lesions strikingly suggest the first stage of acute yellow atrophy. In some cases changes in the thyroid and deficiency of the thyroid secretion have been observed. Some maintain that the fœtus may be the source of the toxin, but no satisfactory evidence has been brought forward. It may be that a placental ferment which has been named syncytrolisin has something to do with the causation of eclampsia, as an extract of human placenta injected into animals has caused convulsions and death.

**A Case of Uterine Inversion occurring Six Days after Delivery.**—Dr. Ferré[6] records a case of inversion of the uterus which occurred six days after delivery. The patient was an unmarried girl of 24 years of age. Labour came on at term and proceeded naturally until the head was on the perineum, when as no further progress was made, the forceps was applied. The birth of the child was followed by a considerable hæmorrhage, and the placenta was therefore expressed. During the four days following labour there was more blood lost than usual. It was noticed that the uterus was not readily defined by palpation, and the distended bladder seemed to reach higher than the fundus uteri. While straining at stool, the patient, without pain, expelled a large mass from the vagina which proved to be the inverted uterus. All efforts to reduce the organ were unavailing, so on the following day it was with considerable difficulty amputated. Septic infection, however, set in and the patient died on the twentieth day of the puerperium.

**Röntgen Rays in Obstetrics and Gynæcology.**—H. Frenner,[7] writing on this subject, says that although the Röntgen rays can have nothing like the importance in obstetrics and gynæcology they bear in the sister science of surgery, yet there are many occasions on which they are of considerable value. Much information can often be gained in the diagnosis of pregnancy, of multiple pregnancy, and of extra-uterine pregnancy. The rays are also of value in the elucidation of presentations and mechanisms, and in the recognition of the form and size of the pelvis. With regard to the question of pregnancy it is only in the second half that the Röntgen rays can help, but they may be of assistance in this latter period in the differential diagnosis between pregnancy and tumour, or in the recognition of pregnancy complicating tumours. Most is to be expected from the rays in the examination of the condition of the bony pelvis. For the examination of malformed children and fœtuses this method has already proved of great service.

**Abdominal Hysterectomy for Severe Concealed Accidental Hæmorrhage.**—J. H. Targett[8] records a very interesting case of concealed accidental hæmorrhage in which he performed abdominal hysterectomy. The patient, a multipara aged 24, had typical signs of concealed accidental hæmorrhage. Previous to admission into hospital she had been given opium, a foot had been brought down and the vagina plugged. On admission into the hospital her condition appeared almost hopeless, and it was considered that abdominal hysterectomy afforded the quickest method of emptying the uterus and controlling the hæmorrhage. The abdomen was opened, the broad ligaments rapidly ligatured on each side, the peritoneum with the bladder pushed down in front, and the uterus removed by supra-vaginal amputation. At the conclusion of the operation the pulse was better than at the beginning. The subsequent progress of the case was by no means uneventful. On the eighth day pus was found in the urine, with some rise of temperature (101°), and thickening and tenderness in the anterior fornix. On the 20th day thrombosis of the left leg developed, and seventeen days later the right leg also became affected. After a prolonged convalescence, recovery ultimately took place.

**Treatment of Puerperal Infection.**—Rudolph Holmes[9] in a paper on this subject, after laying great stress on the importance of prophylaxis, discusses the various curative measures. (1) The douche he considers may be a source of great danger unless properly employed, in that it may introduce organisms from the vagina into the uterus. (2) The curette. This instrument should never be used in the face of an infection, any retained products should be removed with the finger only. (3) Serums. The results of the serum treatment have been disappointing, at present there is no serum on which we can rely. (4) Operative measures. If abscesses form they should be opened and the pus evacuated, but hysterectomy, which has been advocated by some, Holmes considers absolutely unjustifiable. He maintains that puerperal infections are self-limiting diseases, and our treatment should be directed towards supporting the constitutional strength and generally allaying the septic process. Routh[10] strongly recommends the administration of perchloride of iron in these cases, and gives it in doses of 20 minims every two or three hours. Dr. Horrocks[11] says that in his experience the results obtained by the use of antistreptococcic serum were sometimes excellent and sometimes nil; but, as it was impossible to say beforehand whether the serum would do good or not, it was his practice to administer it in 10–20 c.cm. doses two or three times a day. He had seen no bad result follow its use.

[5] Amer. Jour. of Med. Sci., Feb. 1905. [6] Jour. of Obstetrics and Gynæcology of Brit. Empire, May 1905. [7] Deutsch mert Wochnschi, April 27, 1905. [8] Jour. of Obstetrics and Gynæcology of Brit. Empire, May 1905. [9] Birm. Med. Rev. Feb. 1905. [10] The Clin. Jour., Jan. 18, 1905. [11] Brit. Med. Jour., Jan. 14, 1905.

